# Efficacy of Transcranial Magnetic Stimulation and Transcranial Direct-Current Stimulation in Primary Progressive Aphasia Treatment: A Review

**DOI:** 10.3390/brainsci15080839

**Published:** 2025-08-05

**Authors:** Elena Gobbi, Ilaria Pagnoni, Elena Campana, Rosa Manenti, Maria Cotelli

**Affiliations:** Neuropsychology Unit, IRCCS Istituto Centro San Giovanni di Dio Fatebenefratelli, Via Pilastroni, 4, 25125 Brescia, Italy; egobbi@fatebenefratelli.eu (E.G.); ipagnoni@fatebenefratelli.eu (I.P.); rmanenti@fatebenefratelli.eu (R.M.); mcotelli@fatebenefratelli.eu (M.C.)

**Keywords:** repetitive Transcranial Magnetic Stimulation, transcranial Direct-Current Stimulation, language treatment, Primary Progressive Aphasia

## Abstract

**Background**: In recent years, there has been increasing interest in the application of repetitive Transcranial Magnetic Stimulation (rTMS) and transcranial Direct-Current Stimulation (tDCS) to enhance and rehabilitate the language abilities in individuals with neurodegenerative diseases. **Objective**: The aim of this narrative literature review is to investigate the usefulness of rTMS and tDCS to improve language abilities in people with Primary Progressive Aphasia (PPA). **Methods**: This narrative literature review was conducted through a search of the PubMed online database to identify studies investigating the effects of multiple sessions of rTMS or tDCS on language abilities in PPA patients, applied either as stand-alone interventions or in combination with language treatment. **Results**: Thirty-three studies fulfilled the inclusion criteria; five studies employed rTMS without language treatment; two studies applied tDCS as stand-alone intervention; twenty-two studies combined tDCS with language treatment; and four studies assessed the effects of tDCS during verbal task without language treatment. **Conclusions**: rTMS and tDCS applied with or without concomitant language treatment appear to be promising interventions for enhancing language abilities in PPA, with sustained effects reported over time. Further research is necessary to optimise stimulation protocols and to improve our understanding of their long-term effects. Moreover, randomised controlled trials (RCTs) with larger sample sizes are critically needed to clarify the true impact of brain stimulation in PPA, with a focus on changes in cognitive and functional performance, neural activity, and potential molecular correlates.

## 1. Introduction

Primary Progressive Aphasia (PPA) is a neurodegenerative syndrome characterised by a progressive deterioration of language functions while other cognitive domains remain relatively preserved in the early stages of the disease [1,2,3,4,5,6,7]. The onset is insidious, and the disease progressively evolves, resulting in widespread language impairment with an involvement of other cognitive abilities.

PPA is classified into three main variants, each with distinct clinical features, underlying pathology, and neuroimaging findings [8,9,10]: nonfluent/agrammatic (nf/avPPA), semantic (svPPA), and logopenic variants (lvPPA) [2]. The nf/avPPA is primarily characterised by agrammatism in language production and/or effortful, halting speech with sound errors and distortions (apraxia of speech) [1,2,11]. Due to a motor planning deficit, abnormal prosody is often observed [11]. Additional features include impaired comprehension of syntactically complex sentences, whereas single-word comprehension and object knowledge are spared [1,2,7]. Neuroimaging studies in nf/avPPA patients consistently show atrophy in the left inferior frontal gyrus (IFG), left insula, and premotor and supplementary motor areas [1,2,7,12,13]. The svPPA is characterised by a gradual loss of semantic knowledge, which manifests as anomia and single-word comprehension deficits, especially with low-frequency items. Patients with svPPA exhibit fluent spontaneous speech, and word repetition is typically spared, although they may also present surface dyslexia and dysgraphia [1,2,14]. The neuroanatomical profiles typically include bilateral, although mainly left-lateralised, anterior inferior and mesial temporal lobe atrophy [1,2,15]. The lvPPA is primarily characterised by single-word retrieval difficulties and impaired sentence repetition. Furthermore, these patients typically exhibit an absence of frank agrammatism, despite a reduced speech rate and simplified sentence structures; spared motor speech; frequent phonologic errors in spontaneous speech, along with pauses attributable to lexical retrieval difficulties; relatively preserved single-word comprehension and object knowledge [2,16]. Neurodegeneration in lvPPA most commonly affects the left inferior parietal lobule and the left posterior temporal lobe [1,2,16]. Finally, some patients fulfil the core criteria for PPA but do not satisfy the diagnostic features of any established variant, and are therefore defined as “mixed/unclassifiable variant of PPA” [2,17,18]. Regarding the neuropathological substrates of the syndrome, nf/avPPA and svPPA generally belong to the frontotemporal lobar degeneration (FTLD) spectrum, while lvPPA is most often associated with Alzheimer’s disease (AD) pathology [19,20].

PPA is a rare and complex neurodegenerative disorder characterised by considerable clinical heterogeneity. Understanding its epidemiology, clinical features, and progression is crucial for improving diagnostic accuracy and informing treatment strategies. Although epidemiological data on PPA remain limited, studies suggest that it accounts for approximately 0.5–2.5% of neurodegenerative disease cases referred to Memory Clinics, with an incidence of around 1 per 100,000 individuals and an average survival of 8 years [21]. Coyle-Gilchrist et al., 2016 [22] estimated the prevalence of FTLD disorders at 10.84 cases per 100,000 individuals, with an annual incidence of 2.36 cases per 100,000 person-years across Europe. The Salento-Brescia Registry study, conducted in the Italian provinces of Lecce and Brescia, investigated the incidence of FTLD over one year (2017) and identified 63 new cases, resulting in an incidence rate of 3.05 per 100,000 person-years. When standardised to the Italian general population, the incidence was slightly higher at 3.09 per 100,000 person-years [23]. In a subsequent study, Logroscino et al., 2023 [24] assessed the incidence of FTLD across Europe, reporting PPA specific incidence of 0.61 cases per 100,000 person-years. Additionally, a retrospective population-based cohort study conducted in Olmsted County, Minnesota, assessed the incidence of Primary Progressive Apraxia of Speech (PPAOS) and PPA from 2011 to 2022, reporting a PPA incidence of 0.56 cases per 100,000 person-years [25].

PPA is considered an orphan disorder due to its rarity and the associated challenges in research, diagnosis, and treatment. Nevertheless, several non-pharmacological interventions (i.e., speech and language therapy, SLT) have shown promise in symptoms management and in enhancing quality of life for individuals with PPA [26,27,28]. Among these, increasing attention has been directed towards non-invasive brain-stimulation techniques, such as repetitive Transcranial Magnetic Stimulation (rTMS) and transcranial Direct-Current Stimulation (tDCS). These techniques have demonstrated potential in enhancing cognitive functions across both healthy individuals and clinical populations, including those affected by neuropsychiatric disorders, cerebrovascular diseases, neurodegenerative conditions and other cognitive impairments [29,30,31]. Both rTMS and tDCS promote neuroplastic reorganisation by modulating cortical excitability, leading to changes in motor and cognitive functions and are therefore considered useful tools for investigating and enhancing neuroplasticity [31,32,33,34,35,36,37,38,39,40,41,42]. The physiological effects of rTMS and tDCS are influenced by several parameters. Specifically, for tDCS, the effects depend on electrodes size and placement, polarity (with anodal tDCS typically increasing neuronal excitability, while cathodal tDCS decreases it), intensity, duration of the stimulation which influences both the magnitude and maintenance of its effects, the number and frequency of sessions [43,44,45]. Similarly for rTMS, outcomes are influenced by the targeted brain region, the type of coil employed, pulses frequency (low-frequency rTMS generally reduces cortical excitability, while high-frequency rTMS increases it), duration, intensity, the number and frequency of sessions [46,47,48,49,50].

Single sessions of rTMS and tDCS are primarily used to investigate their immediate effects on various cognitive, motor, and mood-related processes, with these changes often being short-lasting, typically persisting for up to an hour post-stimulation [35,51,52,53,54,55,56,57,58]. On the other hand, multiple sessions of rTMS or tDCS tend to produce more significant and lasting effects compared to single session protocols [39,59,60,61,62]. Periodic stimulation protocols, involving multiple sessions, can induce long-term potentiation-like plasticity (LTP), which may persist for days or even weeks. This is achieved by applying a second rTMS or tDCS session during the after-effects of the first session, leading to prolonged increases in cortical excitability [63,64,65]. Accordingly, multiple sessions of rTMS and tDCS have increasingly been employed in research to enhance cognitive functioning and provide therapeutic effects in patients with cognitive impairments by modulating brain networks and promoting synaptic plasticity [61,66,67,68]. Targeting key hubs of higher-level cognitive networks, such as frontoparietal network, has shown promising results [69]. Moreover, combining rTMS and tDCS with cognitive training and exploring their long-term effects seems important for maximising cognitive benefits [70,71]. Combined approaches leverage the neuromodulatory effects of brain stimulation to potentiate the benefits of behavioural therapy, leading to significant and sustained improvements both in cognitive and language outcomes [70,71,72,73,74,75,76,77,78,79,80,81,82,83].

RTMS has shown significant promise in enhancing language outcomes in stroke-related aphasia. Evidence suggests that rTMS can improve naming, repetition, writing, and comprehension abilities in post-stroke aphasia patients [84,85,86,87,88]. tDCS has also been investigated in this population, with anodal stimulation demonstrating potential in improving naming performance [76,89,90,91]. In contrast, the application of rTMS in PPA remain less well established. Preliminary studies suggest that excitatory rTMS, when applied to functionally compromised regions may produce beneficial effects in PPA patients [48]. Similarly, tDCS has yielded some encouraging outcomes too: anodal stimulation over the left IFG has been associated with improved naming accuracy and reduced segmental duration in speech production [75,92,93,94,95,96].

The underlying mechanisms of language impairment differ substantially between stroke-related aphasia and PPA. In stroke-related aphasia, impairment typically results from focal brain damage affecting specific language centres, and recovery mechanisms often involve compensatory recruitment of intact brain regions [97]. This compensatory plasticity represents a key target for rTMS and tDCS interventions, which aim to modulate neural activity and enhance language recovery [98]. Conversely, PPA is characterised by a slowly progressive neurodegeneration process that primarily affects the language-dominant hemisphere. Due to the diffuse and progressive nature of neurodegeneration, compensatory recruitment mechanisms in PPA is less well understood and may not play a significant role as in stroke-related aphasia. These fundamental differences in pathophysiology suggest that responses to neuromodulation techniques, such as rTMS and tDCS, may vary between the two conditions [73,75,76,77,90,91,96,98,99,100].

Both rTMS and tDCS are generally safe and may result in meaningful improvements in language abilities [75,77]. However, findings in PPA remain heterogeneous, reflecting variability in sample characteristics, outcome measures, and stimulation protocols [101]. Notably, there is currently no consensus on optimal stimulation parameters (e.g., site, intensity, duration, number of sessions), which represents a significant challenge in this field and limits the generalisability of results. This variability likely stems from the complex pathophysiological mechanisms underlying PPA, which may differentially influence responsiveness to stimulation, thereby reinforcing the need for more targeted investigations [98,102].

The current body of research suggests that both rTMS and tDCS hold considerable promise as interventions for PPA, particularly when used in conjunction with SLT [73,75,77,79,81,103]. Nonetheless, further research is required to fully understand their mechanisms, optimise treatment protocols, and confirm their efficacy in larger, controlled trials. In particular, tDCS represents a compelling area for continued investigation and potential clinical application in the treatment of PPA.

The rationale for this narrative literature review on rTMS and tDCS interventions in PPA arises from the need to better understand and enhance the effectiveness of treatments for this debilitating condition. Aphasia significantly impacts communication and social participation, making language treatment a crucial aspect of care.

By synthesising the available evidence, the present review aims to provide a comprehensive overview of the application of multiple sessions of rTMS and tDCS in the treatment of PPA, to identify knowledge gaps, and ultimately contribute to improving clinical outcomes for individuals with aphasia associated with neurodegenerative disorders.

## 2. Materials and Methods

### 2.1. Search Strategy

A narrative literature review was conducted to identify studies published in the Medline (PubMed) database, using the following search terms: ‘(transcranial Direct-Current Stimulation OR tDCS OR transcranial Magnetic Stimulation OR TMS) AND (language rehabilitation OR speech therapy OR language treatment OR language training) AND (Primary Progressive Aphasia OR PPA OR semantic dementia)’. The search was limited to English-language publications. The last literature search was carried out on 30 June 2025.

### 2.2. Study Selection Criteria

First, all titles and abstracts were screened independently by two reviewers and duplicates were disregarded. All relevant original research articles were then assessed for inclusion and examined in detail, including their references list, to identify possible additional sources. From this study selection, animal studies and reports based on secondary data, such as meta-analyses, reviews and study protocols, were excluded. Thereafter, the full texts of the remaining articles were read. Studies were considered eligible for inclusion if they met the following criteria:

Inclusion criteria:

1. Original research;
2. Conducted on participants diagnosed with PPA according to the consensus criteria [2];
3. Application of multiple sessions of rTMS or tDCS, with or without language treatment;
4. Inclusion of at least one language outcome measure;
5. Publication before 30 June 2025.

Exclusion criteria:

1. Studies that did not report statistical information on the effects of the intervention (e.g., lack of test statistics, *p*-values, or effect sizes);
2. Studies that included, even partially, the same participants as a main study already selected, without assessing different language outcome measures.

Of 57 full-text articles assessed for eligibility, 33 studies published between 2006 and 2025 fulfilled the inclusion criteria for this narrative literature review. Among these, we identified five studies that employed rTMS without language treatment, two studies that applied tDCS as stand-alone intervention, twenty-two studies that combined tDCS with language treatment and four studies assessed the effects of tDCS during verbal task without language treatment. No studies combining rTMS with language treatment were identified.

**Figure 1:**
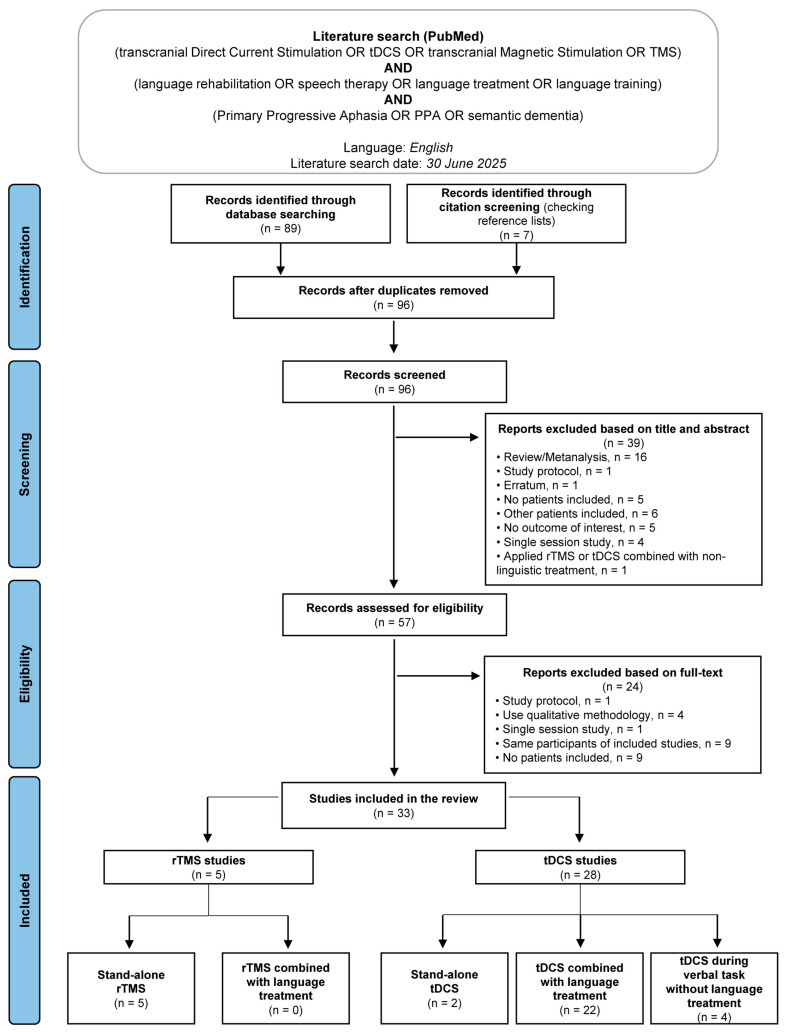
Summary of the literature search—PRISMA 2020 flow diagram.

The PRISMA flow diagram [104], in Figure 1, illustrates the study selection process. The selected studies collectively involved a total of 359 patients with PPA (Table 1, Table 2, Table 3 and Table 4).

## 3. Results

Since the aim of this narrative literature review was to identify studies that investigated the effects of multiple sessions of rTMS or tDCS on language abilities in PPA patients, applied as stand-alone interventions or in conjunction with language treatment, we described the selected studies according to the type of intervention: (a) stand-alone rTMS; (b) stand-alone tDCS; (c) tDCS combined with language treatment; and (d) tDCS during verbal task without language treatment (Table 1, Table 2, Table 3 and Table 4).

### 3.1. Studies That Assessed the Effects of rTMS as a Stand-Alone Intervention

The available evidence suggests that rTMS may be a promising tool for improving language abilities in individuals with PPA. Below is a summary of the main findings from studies that have applied rTMS without concomitant language treatment (Table 1).

The first study to examine the effects of rTMS on language abilities was a crossover trial by Finocchiaro et al., 2006 [105], in which five sessions of high-frequency rTMS were applied over the left prefrontal cortex (PFC) in a single patient with PPA. Compared to placebo, the authors reported a significant improvement in verb production following active stimulation.

Similarly, Trebbastoni et al., 2013 [106] conducted a crossover single-case study applying high-frequency rTMS over the left dorsolateral PFC (DLPFC) in a patient with lvPPA. Results indicated a significant improvement in phonemic fluency and written production tasks after active stimulation, but not following placebo. However, the gains were not maintained one week after treatment completion.

In an uncontrolled single-case study, Bereau et al., 2016 [107] showed that ten sessions of high-frequency rTMS over the left DLPFC led to a significant improvement in verbal fluency in a lvPPA patient, and this benefit was maintained at 12 weeks post-treatment. Additional improvements were observed in attention, executive functioning, and a measure of global cognition, alongside increased cerebral perfusion in the left frontotemporoparietal cortex.

More recently, two studies have investigated the effects of high-frequency rTMS on language abilities in cohorts of PPA patients. Pytel et al., 2021 [108] included two groups: one received ten sessions of active high-frequency rTMS over the left frontal or temporal cortex (using a personalised targeting approach), while the other followed a crossover design (active followed by placebo rTMS, or vice versa). Aggregated data from both active treatment groups showed improvements in spontaneous speech, repetition, reading, and object naming. Benefits were also observed in neuropsychiatric symptoms, as assessed by the Neuropsychiatric Inventory (NPI), and both patients and caregivers reported a positive global impression of change. Post-treatment Positron Emission Tomography (PET) revealed increased metabolism in the left frontal and parieto-temporal regions, as well as in the precuneus and posterior cingulate bilaterally.

Finally, in a parallel randomised controlled trial (RCT), Huang et al., 2023 [109] applied active or placebo high-frequency rTMS over the left DLPFC for right-handed PPA patients or over the right DLPFC for left-handed PPA patients. Significantly greater improvements were reported in oral object naming and overall language functioning in the active group, with effects sustained for up to 24 weeks following treatment. Similar improvements were observed in daily communication, as assessed by the Communicative Activity Log (CAL), with gains maintained up to 12 weeks post-treatment.

### 3.2. Studies That Assessed the Effects of tDCS as a Stand-Alone Intervention

Only a small number of studies have investigated the effects of tDCS applied without language treatment in individuals with PPA (Table 2). Using an RCT, Benussi et al., 2020 [111] evaluated the effects of ten sessions of anodal or placebo tDCS applied over the left PFC in a cohort of PPA patients, reporting significant improvements in phonemic fluency following anodal stimulation. Additional benefits were observed in global cognition, attention, executive functioning, emotion recognition, and neuropsychiatric symptoms (Cambridge Behavioural Inventory, CBI).

Furthermore, Wang et al., 2013 [110] conducted a crossover single-case study in a nf/avPPA patient, who received ten sessions of anodal or placebo tDCS applied over the left posterior perisylvian region (PPR, including Wernicke’s area) in the morning and over the left Broca’s area in the afternoon. The results demonstrated improvements in auditory word comprehension, picture naming, word reading, and word repetition following anodal stimulation. Electroencephalogram (EEG) recordings indicated that performance gains on language tasks were associated with increased activation in Wernicke’s and Broca’s areas, as well as their right-hemisphere homologues and bilateral frontoparietal and parietal regions.

### 3.3. Studies That Assessed the Effects of tDCS Combined with Language Treatment

A substantial number of studies have explored the effects of anodal tDCS combined with language treatment in individuals with PPA (Table 3). These studies are presented below, grouped by study design: RCTs, case series, and single-case.

#### 3.3.1. RCTs Studies

In a parallel RCT, Cotelli et al., 2014 [93] found that ten sessions of anodal tDCS applied over the left DLPFC during an individualised lexical retrieval treatment led to significantly greater improvements in oral naming of both trained and untrained objects, as well as in the oral naming subtest of the Aachener Aphasie Test (AAT), with benefits maintained 12 weeks from baseline. Additional improvements were reported in functional language production abilities, assessed via the Lincoln Speech Questionnaire completed by the caregiver, and in quality of life, measured through the energy subscale of the Stroke and Aphasia Quality-of-Life Scale-39 (SAQOL-39).

In a preliminary study, Tsapkini et al., 2014 [94] reported that both anodal and placebo tDCS conditions led to improvements in written naming/spelling of trained sounds and words, but only the anodal group showed gains in written naming/spelling for untrained items, along with long-lasting benefits for both trained and untrained items up to 8 weeks after the end of treatment. These findings were later confirmed in series of crossover studies that investigated the effects of a specific tDCS protocol involving fifteen sessions of anodal stimulation over the left IFG administered during spelling therapy [95,115,123,134,135,136,137,138,139,140,141]. Tsapkini, et al., 2018 [95] involving thirty-six PPA patients that received oral and written naming/spelling therapy, highlighted a greater effect of anodal tDCS relative to placebo. Subsequently, Wang et al., 2023 [123] re-analysed these data [95] to assess treatment effects on semantic fluency, an additional language outcome measure not previously explored. Their findings revealed that anodal tDCS led to improvements in semantic fluency, which persisted for up to 2 weeks post-treatment. Harris et al., 2019 [115] applied the same protocol in a parallel RCT, reporting that anodal tDCS resulted in significantly greater improvements in oral and written naming/spelling of trained objects, with effects maintained up to 8 weeks following treatment. Additionally, the authors observed reduced GABA levels in the group of PPA patients who received anodal tDCS. The same research group (ClinicalTrials.gov Identifier: NCT02606422) has explored potential predictors of treatment response [134,135,136,137,138,139,140,141]. In this regard, de Aguiar and colleagues [134,135] reported that baseline brain volumes and cognitive profiles predicted treatment efficacy; additionally, Zhao et al., 2021 [141] found that white matter integrity was also a predictor of tDCS outcomes. Moreover, Ficek et al., 2018 [136] investigated that anodal tDCS modulated functional connectivity between the stimulated left IFG and temporal regions, and that these connectivity changes correlated with the magnitude of improvement. Tao et al., 2021 [139] reported that the combined treatment protocol increased the functional segregation of the language system, indicating a normalisation of hyperconnectivity in the left IFG, while Wang, et al., 2024 [140] showed that baseline functional connectivity could predict individual response to treatment. Concerning the individual characteristics as potential predictors of treatment response, Herrmann et al., 2022 [137] found that baseline sleep efficiency modulated treatment-related gains for trained items, and Licata et al., 2023 [138] reported that sex may moderate the effects of anodal tDCS on functional connectivity. Specifically, they observed greater functional connectivity within the default mode network (DMN) in men who received anodal tDCS, and within the language network in women who received the same protocol.

Another crossover study conducted by Roncero et al., 2017 [113] proposed a treatment protocol consisting of ten sessions of either anodal or placebo tDCS applied over the left inferior parieto-temporal region during picture-naming training. The combination of anodal tDCS and language treatment led to greater improvements in oral naming of both trained and untrained objects, with gains maintained at 2-week follow-up from the end of treatment. Additionally, at follow-up, patients showed enhanced performance on a digit span task following anodal tDCS, and informal caregiver reports described behavioural improvements. In a subsequent crossover study exploring the effects of two active tDCS conditions (anodal tDCS over the left parieto-temporal area or anodal tDCS over the left DLPFC) and placebo tDCS, the same authors reported improvements in oral naming of trained objects following both active tDCS conditions. Moreover, generalisation to untrained objects and maintenance of gains at the 2-week follow-up were observed only after anodal tDCS over the left parieto-temporal area [116].

A parallel RCT by Themistocleous et al., 2021 [118] evaluated the effects of anodal or placebo tDCS over the left IFG combined with oral word repetition therapy. The authors reported shorter durations of speech sounds for trained words (both vowels and consonants) at post-treatment in the anodal tDCS group, indicating more efficient and fluent speech production. These improvements generalised to untrained words and were maintained at the 8-week follow-up. Then, a crossover study by Nissim et al., 2022 [121], showed that ten sessions of anodal tDCS over the left frontotemporal region during constraint-induced language therapy (CILT) resulted in significant gains in oral naming, which were maintained up to 6 weeks post-treatment following anodal tDCS compared to placebo. Moreover, baseline measures of cortical thickness and volume were found to predict the degree of tDCS-induced improvement in oral naming. Recently, Borrego-Écija et al., 2023 [122] conducted a crossover RCT involving ten sessions of speech therapy with either anodal or placebo multi-channel high-density tDCS montage, with anodes placed over both frontal and parietal regions of the left hemisphere. The authors did not report a statistically significant effect of anodal tDCS, as patients with PPA in both treatment arms (anodal vs. placebo) showed clinical improvement. However, trends favouring anodal tDCS were observed in semantic association and reading abilities. Functional Magnetic Resonance Imaging (fMRI) analyses revealed increased activity in the right frontal medial cortex and bilateral paracingulate gyrus following anodal tDCS. More recently, Granadillo et al., 2025 [127] conducted a crossover RCT combining anodal or placebo High-Definition tDCS (HD-tDCS) over the left posterior Supramarginal Gyrus (SMG) with non-word repetition and word/non-word reading training. Language gains varied across patients, and no general group-level benefit was observed. However, magnetoencephalography (MEG) and fMRI revealed an excitatory effect of anodal HD-tDCS compared to placebo, suggesting that greater temporoparietal activation and greater functional connectivity were positively associated with language outcomes. Finally, in another crossover RCT, Nickels et al., 2025 [128] examined the effects of phonological treatment during ten sessions of either anodal or placebo tDCS over the left IFG or SMG. Both treatment groups (anodal-first and placebo-first) exhibited significant improvements in phonological transcoding skills; however, participants who received anodal tDCS first showed greater gains in phonological manipulation abilities and positive changes in written narrative production.

Only two crossover RCTs have investigated the effects of anodal tDCS in improving action naming. Fenner et al., 2019 [114] applied between ten and fourteen sessions of either anodal or placebo tDCS over the left IFG during oral and written action naming/spelling treatment, reporting greater improvements in written naming of both trained and untrained actions following anodal stimulation compared to placebo. Furthermore, improvements in written naming of untrained actions were maintained until 8 weeks after treatment. Subsequently, Sheppard et al., 2025 [129] administered fifteen sessions of anodal or placebo tDCS over the left IFG during an action treatment (Verb Network-Strengthening Treatment, VNeST), observing improvements in oral naming of trained actions and in the production of more complete utterances in discourse at post-treatment in both stimulation conditions. A significant advantage of anodal tDCS was reported for oral naming of untrained actions and sentence comprehension, with effects persisting up to the 8-week follow-up. In addition, evidence of generalisation of anodal tDCS effects to sentence production was observed at the end of treatment.

#### 3.3.2. Case-Series and Single-Case Studies

A small number of case-series studies have explored the effects of tDCS administered during language treatment in groups of patients with PPA in the absence of a placebo control condition. Cotelli et al., 2016 [92] demonstrated that ten sessions of anodal tDCS applied over the left DLPFC during an individualised lexical retrieval treatment led to improvements in the oral naming of trained objects. These gains were positively correlated with grey matter volume in the left fusiform gyrus, left middle temporal gyrus, and right inferior temporal gyrus, whereas changes in action naming were associated with grey matter density in the left middle temporal gyrus. Hung et al., 2017 [112] conducted a pre–post study applying ten sessions of anodal tDCS over the left temporoparietal cortex administered during semantic feature analysis. The authors reported improvements in oral naming for trained words, although these gains were not maintained at the 24-week follow-up. Of particular interest, a recent study by George et al., 2025 [126] described improvements in oral naming of trained items, in confrontation naming as measured by the Boston Naming Test (BNT), and in perceived language abilities as reported by caregivers, following twenty sessions of at-home anodal tDCS over the left IFG administered during personalised lexical-retrieval treatment in ten PPA patients.

Single-case studies have also contributed to the understanding of the effects of tDCS when paired with language treatment. At this purpose, Shah-Basak et al., 2022 [117] administered five sessions of HD-tDCS over the left SMG during written naming therapy in a patient with nf/avPPA, using a placebo-controlled crossover design. Improvements in written naming of both trained and untrained objects were observed and remained stable at the 12-week follow-up, regardless of stimulation type. Anodal tDCS additionally promoted normalisation of slowed resting-state MEG activity in the left SMG. In the absence of a placebo control condition, a single-case crossover study conducted by de Aguiar et al., 2022 [119] examined the effects of anodal tDCS over two different regions, combined with letter fluency therapy, in a patient with lvPPA. Anodal tDCS over both the left IFG and inferior parietal lobe (IPL) improved trained and untrained letter fluency, but only stimulation over the IFG resulted in enhanced performance on an object naming task, with longer-lasting effects (up to 8–12 weeks post-treatment). In a crossover study, Nickels et al., 2022 [120] applied ten sessions of anodal or placebo tDCS over the left IFG, paired with phonological treatment in a patient with lvPPA. The intervention led to improvements in phonological transcoding, manipulation, and written sentence construction, with a marked increase in grammatically well-formed and complete written sentences. Benefits emerged following both anodal and placebo stimulation, and were maintained for up to 8 weeks after the end of treatment. The patient and her caregiver also reported positive changes in written communication and self-confidence. In an uncontrolled single-case study, Strunk et al., 2024 [125] administered four phases of ten anodal tDCS sessions over the left anterior temporal lobe during semantic feature analysis treatment in a patient with svPPA. The study reported improvements in oral naming performance, with maintenance of gains for untrained items up to four weeks. A placebo-controlled crossover study by Coemans et al., 2024 [124] included a bilingual lvPPA patient (French: L1; Dutch: L2), who received nine sessions of anodal or placebo tDCS over the right posterolateral cerebellum, combined with semantic and phonological therapy in L2. Anodal tDCS led to significant improvements in oral naming of trained and untrained objects, syntactic comprehension, and repetition of words and nonwords in L2. In L1, improvements in oral naming of untrained objects were observed. At the 8-week post-treatment follow-up, gains in oral naming of trained objects in L2 and untrained objects in L1 were maintained. Following anodal tDCS, enhancement in inhibitory control was also reported and persisted at follow-up.

### 3.4. Studies That Assessed the Effects of tDCS During Verbal Task Without Language Treatment

Four studies applied anodal tDCS across multiple sessions during network activation, without implementing a specific language treatment protocol [130,131,132,133] (Table 4). In an uncontrolled case series, Gervits et al., 2016 [130] administered ten sessions of anodal tDCS over the left frontotemporal region during a narrative task in six PPA patients. The authors reported improvements in speech production, grammatical comprehension, and global language performance, with most of these gains maintained up to 12 weeks post-treatment. In a crossover RCT, McConathey et al., 2017 [131] did not observe a group-level effect of ten anodal or placebo tDCS sessions over the left PFC paired with a narrative task, either at post-treatment or at follow-up assessment. However, they reported greater improvements associated with anodal tDCS in speech repetition among patients with higher baseline performance, and in global language performance, grammatical comprehension, and semantic processing among those with lower baseline scores. In a subsequent crossover RCT, Hosseini et al., 2019 [132] found that ten sessions of anodal or placebo tDCS over the left PFC during a narrative task led to a significant improvement in semantic fluency after anodal tDCS, with effects persisting up to 12 weeks from post-treatment. Finally, Crowley et al., 2024 [133] conducted a crossover study in a lvPPA patient, investigating the effects of two rounds of anodal HD-tDCS administered during a narrative task, targeting the left temporoparietal junction (TPJ) and the left IFG. Anodal stimulation at both sites improved picture description performance; however, only stimulation of the TPJ enhanced confrontation naming. Changes in functional connectivity were also observed beyond the targeted regions.

## 4. Discussion

Primary Progressive Aphasia (PPA) is a neurodegenerative disorder characterised by a gradual decline in language abilities, which substantially impacts individuals’ quality of life, occupational functioning, and social interactions. RTMS and tDCS are non-invasive brain-stimulation techniques that have shown promise in enhancing language recovery in patients with aphasia, a language disorder commonly resulting from stroke or neurodegenerative diseases such as PPA [28,76,90,96,142,143,144].

The aim of this narrative literature review was to provide a comprehensive overview of the application of multiple sessions of rTMS and tDCS in the treatment of PPA, whether applied with or without concomitant language treatment in order to identify knowledge gaps, and ultimately contribute to improving clinical outcomes for individuals with PPA.

Only a small number of studies have investigated the effects of rTMS (n = 5) or tDCS (n = 2) as stand-alone interventions or the effects of tDCS during verbal task without language treatment (n = 4), whereas the majority focused on the effects of tDCS paired with language treatment (n = 22), indicating a growing interest in these combined treatment protocols. Most of the included studies employed a crossover RCT design (39%), followed by single-case studies (30%), parallel RCTs (18%), and case series (12%) (Table 1, Table 2, Table 3 and Table 4).

Focusing on rTMS, all included studies applied high-frequency (10–20 Hz) rTMS without the inclusion of concurrent language treatment. Despite variability in the number of sessions (ranging from 5 to 20) and the PPA variants included, all studies reported a significant positive effect on language abilities immediately after rTMS application [105,106,107,108,109]. Furthermore, generalisation effects were assessed in all rTMS studies, although only two reported improvements in clinical and/or cognitive outcomes [107,108]. In addition, three of the five studies evaluated long-term effects of stimulation (ranging from 1 to 24 weeks), and two reported sustained gains lasting up to 12 or 24 weeks [107,109] (Table 1).

With regard to tDCS, all studies employed anodal stimulation. Two studies administered anodal tDCS as a stand-alone intervention, both reporting improvements in language abilities following stimulation [110,111], with one also demonstrating generalisation to clinical and cognitive outcomes [111] (Table 2).

The twenty-two studies that combined tDCS with language treatment varied in terms of PPA patient samples (variant, language background, severity, etc.), number of sessions (ranging from 5 to 40), tDCS intensity (1–2 mA; for high density tDCS, the maximum current delivered through all the electrodes was 4 mA), tDCS session duration (20–30 min), electrode placement, and type of language treatment administered. Notably, in one of these studies, participants received at-home tDCS [126]. Despite this heterogeneity, the majority of studies (approximately 91%) reported positive effects of the combined protocol (anodal tDCS plus language treatment) on at least one language outcome at the end of treatment. Specifically, all studies assessing performance on trained language stimuli observed improvements after anodal tDCS paired with language treatment [36,92,93,94,95,113,114,115,116,117,118,119,120,121,122,124,125,126,128,129]. Most of the included studies (91%) also examined potential generalisation effects; among these, 75% reported effects to untrained stimuli and/or other language tasks [92,93,94,95,113,114,117,118,119,122,123,124,125,126,129], while fewer studies (15%) found evidence of generalisation to clinical or cognitive outcome measures [93,113,128]. Furthermore, long-term effects (ranging from 2 to 24 weeks) were evaluated in almost all studies (95%), and all of them (95%) reporting maintenance of treatment gains at follow-up intervals between 2 and 12 weeks [92,93,94,95,113,114,115,116,117,118,119,120,121,122,123,124,125,127,128,129] (Table 3). Finally, four studies investigated the application of tDCS during verbal (narrative) task without language treatment [130,131,132,133], and most of these reported improvements in language performance [130,132,133], with gains maintained up to 12 weeks follow-up [130,132] (Table 4).

An interesting and potentially clinically feasible approach involves the stimulation of cortical areas known to be functionally related to the language network. The rationale for selecting specific regions for rTMS and tDCS in aphasia and PPA typically entails targeting regions within the language network that may facilitate rebalancing of interhemispheric activity, enhance cortical excitability in the affected hemisphere, and inhibit maladaptive plasticity in the non-affected hemisphere [98,102].

As highlighted in this review, most studies have targeted frontal regions (PFC, IFG, DLPFC) [92,93,94,95,105,106,107,109,110,111,114,115,116,118,119,120,121,122,123,126,128,129,130,131,132,133]. These areas play a key role in language production, cognitive control, and network integration. Stimulation of these regions has yielded promising outcomes in improving both language and cognitive functions, supporting their selection as primary targets for neuromodulation in PPA. Additionally, temporo-parietal areas have emerged as valuable targets, owing to their involvement in modulating interhemispheric inhibition, enhancing cortical excitability, and promoting neuroplasticity [112,113,116,117,119,122,125,127,128,133]. Finally, a growing body of evidence supports the cerebellum as a novel and promising site for stimulation. Its involvement in language processing, its capacity to induce neuroplastic changes, the safety profile of non-invasive stimulation techniques, and recent findings from clinical trials collectively justify its inclusion in neuromodulatory treatment protocols for PPA [124,145,146,147,148,149,150,151,152].

In summary, both rTMS and tDCS applied with or without concomitant language treatment, appear to be promising interventions for enhancing language abilities in PPA, with sustained effects reported over time. Specifically, tDCS paired with language treatment has emerged as the most widely used intervention in the literature, consistently demonstrating significant short- and long-term improvements in language abilities.

Promising evidence has emerged from this review; however, the literature on the efficacy of rTMS and tDCS in improving language functions in PPA remains inconsistent and is marked by several methodological and clinical challenges. Some studies report significant improvements in language abilities, such as naming, verb production, and speech fluency [29,96], while others have found no significant effects or only temporary benefits [77,101,142]. These variable outcomes are likely influenced by a range of factors, including differences in study design, sample sizes, stimulation parameters, the presence or absence of concomitant behavioural interventions, outcome measures, and the specific PPA variants investigated. This heterogeneity makes it difficult to draw firm conclusions and complicates direct comparisons across studies [28]. Although case studies represent a valuable tool for evaluating treatment effectiveness, especially in real-world settings and for rare conditions, their findings should be interpreted with caution due to inherent limitations, such as the absence of randomisation and potential for bias. They are best used in conjunction with other study designs, including RCTs and meta-analyses, to provide a more comprehensive understanding of treatment effectiveness [153,154].

In addition, individual characteristics of patients, such as gender and age, may play a crucial role in the effectiveness of neuromodulatory interventions. Gender-related differences in cortical anatomy, as well as age-related changes in brain structure and function underscore the importance of personalised approaches to optimise the efficacy of rTMS and tDCS. These variables, encompassing anatomical, hormonal, and neurophysiological factors, can significantly influence the outcomes of rTMS and tDCS, and should therefore be carefully considered when tailoring rTMS and tDCS stimulation protocols to maximise therapeutic benefits for each individual [136,138,155,156,157,158,159,160,161].

Emerging evidence suggests that individual factors may significantly influence treatment outcomes. For instance, a recent meta-analysis reported that women with PPA tend to achieve greater improvements in language abilities compared to men [77]. Licata et al., 2023 [138] found that women exhibited greater responsiveness to anodal/active tDCS over left-hemisphere language-related regions, potentially attenuating the decline in processing efficiency. Furthermore, the study reported enhanced responsiveness to active-tDCS stimulation in men, alongside baseline differences in functional connectivity within the default mode network between sexes, suggesting distinct neural mechanisms underlying stimulation responsiveness in men and women with PPA.

Despite growing interest in patient-specific factors, personalised stimulation protocols remain underexplored and none of the included studies systematically examined these variables, revealing a critical gap in the current literature.

Future research should aim to optimise cortical targets based on their involvement in the language network, integrate neuromodulation with behavioural therapies, and personalise intervention protocols by considering individual factors, such as age and gender, that influence treatment responsiveness. Additionally, larger and well-controlled trials are necessary to clarify the long-term efficacy of these interventions.

## 5. Conclusions

RTMS and tDCS represent promising therapeutic interventions for language rehabilitation in PPA. Findings from the reviewed studies suggest that these non-invasive brain-stimulation techniques, particularly when combined with language treatment, may enhance the effectiveness of conventional speech and language interventions. However, further research is necessary to optimise stimulation protocols and improve our understanding of their long-term effects. Moreover, RCTs with larger sample sizes are critically needed to clarify the true impact of brain stimulation in PPA, with a focus on changes in cognitive and functional performance, neural activity, and potential molecular correlates [162].

## Figures and Tables

**Table 1:** rTMS as a stand-alone intervention.

Study	Patients	Age: Mean (SD); Sex: M/F	Protocol Design	rTMS (Number of Sessions, Target Area, and Parameters)	Placebo	Follow-Up	Outcome Measures		Results	
							Language	Clinical and Cognitive	Language	Clinical and Cognitive
Finocchiaro et al., 2006 [105]	1 PPA *	60; M	Single-case crossover	- Sessions: ~5 min/session, 1 daily session, 5 sessions/1 week - Target area: left PFC, 6 cm anterior and 1 cm ventral from the motor spot - Frequency: 10 trains of 20 Hz (40 pulses per train, 30 s inter-train intervals) - Intensity: 90% MT - rTMS device: Cadwell, figure-of-eight coil	Yes	None	- Sentence-completion (verb) - Sentence-completion (noun or determiner)	- Memory span (pseudo-words and numbers)	↑ Sentence-completion (verb) after active rTMS	No significant improvements
Trebbastoni et al., 2013 [106]	1 lvPPA	50; M	Single-case crossover	- Sessions: 20 min/session, 1 daily session, 5 sessions/1 week - Target area: left BA44 and left BA45 - Frequency: 30 trains of 20 Hz (50 pulses per train) - Intensity: 100% MT - rTMS device: Magstim Rapid ^2^, Brainsway’s H-coil	Yes	1 week post-treatment	- Phonemic fluency - Written production	- MMSE - FAB - CDT - BDT	↑ Phonemic fluency after active rTMS ↑ Written production after active rTMS Gains not maintained at **follow-up**	No significant improvements
Bereau et al., 2016 [107]	1 lvPPA	66; F	Single-case pre-post	- Sessions: 20 min/session, 2 daily sessions, 10 sessions/1 week - Target area: left DLPFC, 5 cm anterior, parasagittal line from the hand motor area - Frequency: 40 trains of 10 Hz (50 pulses per train, 25 s inter-train intervals) - Intensity: 100% MT - rTMS device: Magstim Super rapid, figure-of-eight coil	No	12 weeks post-treatment	- Sentence Comprehension - Picture naming - Word, non-word, sentence repetition - Isaac Set Test - Phonemic fluency - Semantic fluency	- MMSE - MDRS - Crossing-Off Test - TMT, part A - Stroop - FCSRT - Digit span FW and BW - DMS48	↑ Phonemic fluency ↑ Semantic fluency Gain maintained at **follow-up**: ↑ Semantic fluency	↑ MMSE ↑ TMT, part A ↑ Stroop
Pytel et al., 2021 [108]	14 nf/avPPA 6 svPPA	66.9 (7.2); 8/12	Case series pre-post (13 PPA) Crossover RCT (7 PPA)	- Sessions: 1 daily session, 15 sessions/3 weeks - Target areas: Left: IFG, SFG, DLPFC and anterior temporal lobe; Right: SFG - Frequency: Trains of 20 Hz (1500 pulses per session, 20 s inter-train intervals) - Intensity 100% MT - rTMS device: Magstim Rapid ^2^, figure-eight coil	Yes, on the RCT	None	- Spontaneous speech - Story Reading - Object naming - Repetition - Picture semantic association (only svPPA)	- ACE-III - Perception of change reported by the patient and caregiver - NPI	↑ Spontaneous speech after active rTMS ↑ Story Reading after active rTMS ↑ Oral object naming after active rTMS ↑ Repetition after active rTMS	↑ NPI (total score, depression and apathy subscores) after active rTMS ↑ Perception of change reported by the patient and caregiver after active rTMS
Huang et al., 2023 [109]	16 nf/avPPA 12 svPPA 12 lvPPA	n.a.; 19/21	Parallel RCT	- Sessions: ~3 min/session, 1 daily session, 20 sessions/4 weeks - Target area: F3 for right-handed patients, F4 for left-handed patients - Frequency: 50 trains of 10 Hz (20 pulses per train, 2 s inter-train intervals) - Intensity: 120% MT - rTMS device: Magstim, figure-of-eight coil	Yes	12 and 24 weeks post-treatment	- BNT - Spontaneous speech, auditory-verbal comprehension, repetition, naming, reading, writing, apraxia and aphasia quotient from WAB - CAL	- HAMA - HRDS	↑ BNT in both groups (active > placebo) ↑ WAB—aphasia quotient in both groups (active > placebo) ↑ CAL in both groups (active > placebo) Gain maintained at 12 weeks **follow-up**: ↑ CAL in both groups (active > placebo) Gains maintained at 24 weeks **follow-up:** ↑ BNT in both groups (active > placebo) ↑ WAB—aphasia quotient in both groups (active > placebo)	No significant improvements

* PPA subtype was not specified in the original study; ↑: Statistically significant improvement; ACE-III: Addenbrooke’s Cognitive Examination; BA: Broadmann Area; BDT: Block Design Test; BNT: Boston Naming Test; BW: Backward; CAL: Communicative Activity Log; CDT: Clock Drawing Test; cm: centimetres; DLPFC: Dorsolateral Prefrontal Cortex; DMS48: Delayed Matching-to-Sample-48 items; F: female; FAB: Frontal Assessment Battery; FCSRT: Free and Cued Selective Reminding Test; FW: Forward; HAMA: Hamilton Anxiety Scale; HRDS: Hamilton Rating Scale for Depression; Hz: Hertz; IFG: inferior frontal gyrus; lvPPA: logopenic variant of Primary Progressive Aphasia; M: male; MDRS: Mattis Dementia Rating Scale; MMSE: Mini Mental State Examination; MT: motor threshold; n.a.: not available; nf/avPPA: non-fluent/agrammatic variant of Primary Progressive Aphasia; NPI: Neuropsychiatric Inventory; PFC: Prefrontal Cortex; PPA: Primary Progressive Aphasia; RCT: Randomised Controlled Trial; rTMS: repetitive Transcranial Magnetic Stimulation; SD: Standard Deviation; SFG: superior frontal gyrus; svPPA: semantic variant of Primary Progressive Aphasia; TMT: Trail Making Test; WAB: Western Aphasia Battery.

**Table 2:** tDCS as a stand-alone intervention.

Study	Patients	Age: Mean (SD); Sex: M/F	Protocol Design	tDCS (Number of Sessions, Montage, and Parameters)	Placebo	Follow-Up	Outcome Measures		Results	
							Language	Clinical and Cognitive	Language	Clinical and Cognitive
Wang et al., 2013 [110]	1 nf/avPPA	67.0; F	Single-case crossover	- Sessions: 20 min/session, 2 daily sessions, 10 sessions/5 days - Montage: anode over left PPR (morning) and left Broca’s area (afternoon), cathode over shoulder - Electrodes size: anode 4.5 × 5.5 cm, cathode 4.5 × 5.5 cm. - Intensity: 1.2 mA - tDCS device: IS200	Yes	None	- Auditory word-picture identifications from PACA - Picture naming from PACA - Oral word reading from PACA - Word repetition from PACA	None	↑ Auditory word-picture identifications from PACA after anodal tDCS, ↑ Picture naming from PACA after anodal tDCS ↑ Oral word reading from PACA after anodal tDCS ↑ Word repetition from PACA after anodal tDCS	-
Benussi et al., 2020 [111]	30 PPA *	n.a.	Parallel RCT	- Sessions: 20 min/day, 1 daily session, 10 sessions/2 weeks - Montage: anode over left PFC, cathode over right deltoid muscle - Electrodes size: anode 5 × 7 cm, cathode 5 × 7 cm - Intensity: 2 mA	Yes	12 and 24 weeks from baseline	- Phonemic fluency	- MMSE - TMT - Stroop - Digit symbol - modified Ekman emotion recognition - CBI	↑ Phonemic fluency in anodal tDCS group Gains maintained at **follow-ups**: - n.a. for PPA group	↑ MMSE in anodal tDCS group ↑ TMT in anodal tDCS group ↑ Digit symbol in anodal tDCS group ↑ modified Ekman emotion recognition in anodal tDCS group ↑ CBI in anodal tDCS group Gains maintained at **follow-ups**: - n.a. for PPA group

* PPA subtype was not specified in the original study; ↑: Statistically significant improvement; CBI: Cambridge Behaviour Inventory; cm: centimetres; F: female; M: male; mA: milliAmpere; MMSE: Mini Mental State Examination; n.a.: not available; nf/avPPA: non-fluent/agrammatic variant of Primary Progressive Aphasia; PACA: Psycholinguistic Assessment in Chinese Aphasia; PFC: Prefrontal Cortex; PPA: Primary Progressive Aphasia; PPR: Posterior Perisylvian Region; RCT: Randomised Controlled Trial; SD: Standard Deviation; tDCS: transcranial Direct-Current Stimulation; TMT: Trail-Making Test.

**Table 3:** tDCS combined with language treatment.

Study	Patients	Age: Mean (SD); Sex: M/F	Protocol Design	tDCS (Number of Sessions, Montage, and Parameters)	Language Treatment	Placebo	Follow-Up	Outcome Measures		Results	
								Language	Clinical and Cognitive	Language	Clinical and Cognitive
Cotelli et al., 2014 [93]	16 nf/avPPA	66.9 (8.2); 6/10	Parallel RCT	- Sessions: 25 min/day, 1 daily session, 10 sessions/2 weeks - Montage: anode over left DLPFC (BA8/9), cathode over right arm - Electrodes size: anode 5 × 5 cm, cathode 6 × 10 cm - Intensity: 2 mA - tDCS device: BrainStim	Individualised lexical retrieval treatment	Yes	12 weeks from baseline	- Phonemic fluency - Semantic fluency - AAT - Naming and sentence comprehension from BADA - Oral naming of trained objects - Oral naming of untrained objects - Action naming - Lincoln Speech Questionnaire - ASRS	- MMSE - RCPM - Story Recall - ROCF - Digit span - TMT - SAQOL-39	↑ Naming from AAT in anodal tDCS group ↑ Oral naming of trained items in both groups (anodal > placebo) ↑ Oral naming of untrained items in both groups ↑ Lincoln Speech Questionnaire (production abilities) in anodal tDCS group Gains maintained at **follow-up**: ↑ Naming from AAT in anodal tDCS group ↑ Oral naming of trained items in both groups ↑ Oral naming of untrained items in both groups	↑ SAQOL-39 (energy subscale) in anodal tDCS group Gains not maintained at **follow-up**
Tsapkini et al., 2014 [94]	2 nf/avPPA 4 lvPPA	n.a.; 3/3	Crossover RCT	- Sessions: 20 min/day, 1 daily session, 15 sessions/3–5 weeks - Montage: anode over left IFG (F7) - Electrodes size: anode 2 × 2 inch, cathode 2 × 2 inch - Intensity: 1–2 mA - tDCS device: Chattanooga Ionto	Spelling therapy	Yes	2 and 8 weeks post-treatment	- Written naming/spelling of trained sounds and words - Written naming/spelling of untrained sounds and words	None	↑ Written naming/spelling of trained items after both conditions ↑ Written naming/spelling of untrained items after anodal tDCS Gains maintained at **follow-ups**: ↑ Written naming/spelling of trained items after anodal tDCS ↑ Written naming/spelling of untrained items after anodal tDCS	-
Cotelli et al., 2016 [92]	18 nf/avPPA	66.5 (9.5); 9/9	Case series pre-post	- Sessions: 25 min/day, 1 daily session, 10 sessions/2 weeks - Montage: anode over left DLPFC, cathode on right arm - Electrodes size: anode 5 × 5 cm, cathode 6 × 10 cm - Intensity: 2 mA - tDCS device: BrainStim	Individualised lexical retrieval treatment	No	12 weeks from baseline	- Phonemic fluency - Semantic fluency - AAT - Sentence comprehension - Oral naming of trained objects - Oral naming of untrained objects - Action naming - Lincoln Speech Questionnaire - ASRS	- MMSE - RCPM - Story Recall - ROCF - Digit span - TMT - SAQOL-39	↑ Naming from AAT ↑ Oral naming of trained items ↑ Oral naming of untrained items Gains maintained at **follow-up**: ↑ Oral naming of trained items ↑ Oral naming of untrained items	No significant improvements
Hung et al., 2017 [112]	3 svPPA 1 lvPPA	68.3 (7.7); 2/2	Case series pre-post	- Sessions: 20 min/day, 1 daily session, 10 sessions/2 weeks - Montage: Anode over left temporoparietal region (P3), cathode on forehead - Electrodes size: 1 cm^2^ electrode within 5 cm^2^ sponges - Intensity: 1.5 mA - tDCS device: Magstim Eldith	Semantic feature analysis therapy	No	24 weeks post-treatment	- Oral naming of trained words - Oral naming of untrained words	None	↑ Oral naming of trained items Gain not maintained at **follow-up**	-
Roncero et al., 2017 [113]	6 nf/avPPA 2 svPPA 2 lvPPA	67.4 (5.9); 7/3	Crossover RCT	- Sessions: 30 min/session, 1 daily session, 10 session/18 days - Montage: anode over left inferior parieto-temporal lobe (P3), cathode over right fronto-orbital - Electrodes size: anode 5 × 7 cm, cathode 5 × 7 cm - Intensity: 2 mA - tDCS device: NeuroConn DC Stimulator plus	Picture naming training	Yes	2 weeks post-treatment	- Oral naming of trained objects - Oral naming of untrained objects - Phonemic fluency - Semantic fluency	- Digit span FW and BW - MoCA - MMSE - Informal caregiver interview	↑ Oral naming of trained items after both conditions (anodal > placebo) ↑ Oral naming of untrained items after anodal tDCS Gains maintained at **follow-up**: ↑ Oral naming of trained items after anodal tDCS ↑ Oral naming of untrained items after anodal tDCS	Perception of positive change after anodal tDCS Gain at **follow-up**: Higher performance in Digit span after anodal tDCS
Tsapkini et al., 2018 [95]	14 nf/avPPA 10 svPPA 12 lvPPA	nf/avPPA: 70.0 (5.8); 9/5 svPPA: 68.6 (5.2); 5/5 l/phvPPA: 65.3 (8.4); 6/6	Crossover RCT	- Sessions: 20 min/day, 1 daily session, 15 sessions/3 weeks - Montage: anode over left IFG (F7), cathode over right cheek - Electrodes size: anode 5 × 5 cm, cathode 5 × 5 cm - Intensity: 2 mA - tDCS device: Soterix Clinical Trials Model 1500	Oral and written objects naming/spelling therapy	Yes	2 and 8 weeks post-treatment	- Written naming/spelling of trained objects - Written naming/spelling of untrained objects	None	↑ Written naming/spelling of trained items after both conditions (anodal > placebo) ↑ Written naming/spelling of untrained items after both conditions (anodal > placebo) Gains maintained at **follow-ups**: ↑ Written naming/spelling of trained items after anodal tDCS ↑ Written naming/spelling of untrained items after anodal tDCS	-
Fenner et al., 2019 [114]	6 nf/avPPA 5 lvPPA	69.2 (5.9); 7/4	Crossover RCT	- Sessions: 20 min/day, 1 daily session, 10–14 days/2–3 weeks - Montage: Anode over left IFG (F7), cathode over right cheek - Electrodes size: anode 5 × 5 cm, cathode 5 × 5 cm - Intensity: 2 mA - tDCS device: Soterix Clinical Trials Model 1500	Oral and written action naming/spelling therapy	Yes	2 and 8 weeks post-treatment	- Written naming/spelling of trained actions - Written naming/spelling of untrained actions	None	↑ Written naming/spelling of trained items after both conditions (anodal > placebo) ↑ Written naming/spelling of untrained items after both conditions (anodal > placebo) Gains maintained at **follow-ups**: ↑ Written naming/spelling of untrained items after both conditions (anodal > placebo)	-
Harris et al., 2019 [115]	10 nf/avPPA 6 svPPA 6 lvPPA	66.9 (7.5); 11/11	Parallel RCT	- Sessions: 20 min/day, 1 daily session, 15 sessions/3 weeks - Montage: anode over left IFG (F7), cathode over right cheek - Electrodes size: anode 2 × 2 inch, cathode 2 × 2 inch - Intensity: 2 mA - tDCS device: Soterix Clinical Trials Model 1500	Oral and written objects naming/spelling therapy	Yes	8 weeks post-treatment	- Oral and written naming/spelling of trained objects	None	↑ Oral and written naming/spelling of trained items in both groups (anodal > placebo) Gains maintained at **follow-up**: ↑ Oral naming/spelling of trained items in both groups (anodal > placebo)	-
Roncero et al., 2019 [116]	4 nf/avPPA 4 svPPA 4 lvPPA	65.4 (6.0); 8/4	Crossover RCT	- Sessions: 30 min/day, 1 daily session, 10 sessions/3 weeks - Montage: anode over left parieto-temporal area (TP9), cathode over right fronto-orbital area, or anode over left DLPFC (F3), cathode over right deltoid muscle - Electrodes size: anode 5 × 7 cm, cathode 5 × 7 cm - Intensity: 2 mA - tDCS device: NeuroConn DC Stimulator MC	Picture naming training	Yes	2 and 8 weeks post-treatment	- Oral naming of trained objects - Oral naming of untrained objects - Phonemic fluency - Semantic fluency	- Digit span FW and BW - MoCA - MMSE	↑ Oral naming of trained items after all conditions (anodal conditions > placebo) Gains at 2 weeks **follow-up**: ↑ Oral naming of trained items after anodal left parieto-temporal tDCS ↑ Oral naming of untrained items after anodal left parieto-temporal tDCS Gains not maintained at 8 weeks **follow-up**	No significant improvements
Shah-Basak et al., 2022 [117]	1 nf/avPPA	67.0; M	Single-case crossover	- Sessions: 20 min/day, 1 daily session, 5 sessions/1 weeks - Montage: HD-tDCS, 3 × 1 centre-surround electrode, anode over left SMG - Intensity: 2 mA - HD-tDCS device: NeuroConn DC stimulator MC	Written naming therapy	Yes	12 weeks	- Written naming of trained objects - Written naming of untrained objects	None	↑ Written naming of trained items after both conditions ↑ Written naming of untrained items after both conditions Gains maintained at **follow-up**: ↑ Written naming of trained items after both conditions ↑ Written naming of untrained items after both conditions	-
Themistocleous et al., 2021 [118]	8 nf/avPPA with AOS	66.0 (8.3); 4/4	Parallel RCT	- Sessions: 20 min/day, 1 daily session, 15 sessions/3 weeks - Montage: anode over left IFG (F7), cathode over right check - Electrodes size: anode 5 × 5 cm, cathode 5 × 5 cm - Intensity: 2 mA - tDCS device: Soterix Clinical Trials Model 1500	Oral word repetition therapy	Yes	8 weeks post-treatment	- Sound duration for trained words - Sound duration for untrained words - Vowel duration for trained words - Vowel duration for untrained words - Consonant duration for trained words - Consonant duration for untrained words	None	↑ Sound duration for trained and untrained items in anodal tDCS group ↑ Vowel duration for trained and untrained items in anodal tDCS group ↑ Consonant duration for trained and untrained items in anodal tDCS group Gains maintained at **follow-up**: ↑ Sound duration for trained items in anodal tDCS group ↑ Vowel duration for trained items in anodal tDCS group	-
de Aguiar et al., 2022 [119]	1 lvPPA	72.0; M	Single-case crossover	- Sessions: 20 min/day, 1 daily session, 10 sessions/2 weeks - Montage: Anode over left IFG (F7) or left IPL (TP3), cathode over right cheek - Electrodes size: anode 5 × 5 cm, cathode 5 × 5 cm - Intensity: 2 mA	Letter fluency therapy	No	2 and 8 or 2 and 12 weeks post-treatment	- Letter fluency for trained letters (number of words) - Letter fluency for untrained letters (number of words) - Oral objects naming	None	↑ Letter fluency for trained items after both conditions ↑ Letter fluency for untrained items after both conditions ↑ Oral objects naming after anodal left IFG Gains maintained at 2 weeks **follow-up**: ↑ Letter fluency for trained items after both conditions ↑ Letter fluency for untrained items after both conditions Gains maintained at 8 or 12 weeks **follow-ups**: ↑ Letter fluency for trained items after anodal left IFG tDCS ↑ Oral objects naming after anodal left IFG	-
Nickels et al., 2022 [120]	1 lvPPA	71.0; F	Single-case crossover	- Sessions: 20 min/day, 1 daily session, 10 sessions/2 weeks - Montage: anode over left IFG (F5), cathode over right supraorbital location (FP2) - Electrodes size: anode 5 × 7 cm, cathode 5 × 7 cm - Intensity: 1.5 mA - tDCS device: NeurConn DC Stimulator Plus	Phonological treatment	Yes	8 weeks post-treatment	- Blending sounds for words and non-words - Reading non-words - Phonological manipulation (APB) - Letter-sound transcoding - Sound-letter transcoding - Aphasia quotient from WAB - BNT - Semantics Camel and Cactus test - Reading words and non-words - Spelling words and non-words - Rainbow passage - Written narratives skills - Repetition of word and non-word - Perception of change reported by the participant and by the caregiver	- Digit span FW and BW - Recognition of faces from Warrington memory test	↑ Blending sounds for words after anodal tDCS ↑ Blending sounds for non-words after both conditions ↑ Reading non-words after anodal tDCS ↑ Phonological manipulation after both conditions ↑ Sound-letter transcoding after anodal tDCS ↑ Letter-sound transcoding after placebo tDCS ↑ Spelling non-words after anodal tDCS ↑ Written narratives skills after both conditions Perception of positive change reported by the participant and the caregiver after both conditions Gains maintained at **follow-up**: ↑ Blending sounds for words after anodal tDCS ↑ Reading non-words after anodal tDCS ↑ Phonological manipulation from APB after both conditions ↑ Sound-letter transcoding after anodal tDCS ↑ Spelling words and non-words after both conditions ↑ Written narratives after both conditions	No significant improvements
Nissim et al., 2022 [121]	2 nf/avPPA 1 svPPA 9 lvPPA	66.9 (6.4); 8/4	Crossover RCT	- Sessions: 20 min/day, 1 daily session, 10 sessions/2 weeks - Montage: HD-tDCS, 1 × 1 transcranial DC and 4 × 1 Multi-Channel Stimulation Interface; anode over left frontotemporal region (FT7), surrounding cathodes over F7, T7, FC5, FT9 - Intensity: 1.5 mA - HD-tDCS device: Soterix Medical	Constraint-induced language therapy	Yes	6 weeks post-treatment	- Spontaneous speech, auditory-verbal comprehension, repetition and naming from WAB-R	None	↑ Naming from WAB-R after anodal tDCS Gains maintained at **follow-up**: ↑ Naming from WAB-R after anodal tDCS	-
Borrego-Écija et al., 2023 [122]	6 nf/avPPA 4 svPPA 5 lvPPA	63.0 (8.4); 5/10	Crossover RCT	- Sessions: 26 min/day, 1 daily session, 10 sessions/2 weeks - Montage: multi-channel high-density tDCS with seven electrodes over left frontal and parietal regions vertex (C1, F7, FC1, 19 FC5, Fpz, P7, and PO8) - Electrodes size: 1 cm radius - Intensity: max 4 mA - tDCS device: StarStim	Speech therapy	Yes	4 and 12 weeks post-treatment	- Phonemic fluency of trained letters - Phonemic fluency of untrained letters - Semantic fluency of trained semantic categories - Semantic fluency of untrained semantic categories - Oral naming of trained objects - Oral naming of untrained objects - Comprehension of trained single-word - Comprehension of untrained single-word - Trained semantic association - Untrained semantic association - Trained reading speed - Untrained reading speed	None	↑ Composite score of all evaluated tasks after both tDCS conditions Gains maintained at 4 weeks **follow-up**: ↑ Composite score of all evaluated tasks after both tDCS conditions Gains not maintained at 12 weeks **follow-up**	-
Wang et al., 2023 [123]	13 nf/avPPA 9 svPPA 14 lvPPA	nf/avPPA: 69.8 (6.0); 8/5 svPPA: 67.9 (5.0); 4/5 l/phvPPA: 66.3 (8.1); 7/7	Parallel RCT	- Sessions: 20 min/day, 1 daily session, ~12 sessions/3 weeks - Montage: anode over left IFG (F7), cathode over right cheek - Electrodes size: anode 5 × 5 cm, cathode 5 × 5 cm - Intensity: 2 mA - tDCS device: Soterix 1 × 1 Clinical Trials	Oral and written objects naming/spelling therapy	Yes	2 and 8 weeks post-treatment	- Phonemic fluency - Semantic fluency	- Digit span FW and BW - TMT - PHQ-9	↑ Semantic fluency in anodal tDCS group Gain maintained at 2 weeks **follow-up**: ↑ Semantic fluency in anodal tDCS group Gain not maintained at 8 weeks **follow-up**	No significant improvements
Coemans et al., 2024 [124]	1 bilingual lvPPA (L1: French; L2: Dutch)	59.0; M	Single-case crossover	- Sessions: 20 min/day, 1 daily session, 9 sessions/3 weeks - Montage: anode over right posterolateral cerebellum, cathode over right deltoid muscle - Electrodes size: anode 3 × 3 cm, cathode 3 × 3 cm - Intensity: 2 mA - tDCS device: Oasis Pro	Semantic and phonological speech and language therapy provided in L2	Yes	8 weeks post-treatment	- Oral naming of trained objects in L2 - Oral naming of untrained objects in L1 and L2 - BAT in L1 and L2 - Picture Description in L1 and L2 - Cookie Theft in L1 and L2	- MMSE - ANT - Stroop	↑ Oral naming of trained items in L2 after anodal tDCS ↑ Oral naming of untrained items in L1 and L2 after anodal tDCS ↑ Syntactic comprehension, Repetition of words and nonsense words from BAT in L2 after anodal tDCS Gains maintained at **follow-up**: ↑ Oral naming of trained items in L2 after anodal tDCS ↑ Oral naming of untrained items in L1 after anodal tDCS	↑ ANT after anodal tDCS Gains maintained at **follow-up**: ↑ ANT after anodal tDCS
Strunk et al., 2024 [125]	1 svPPA	56.0; F	Single-case pre-post	- Sessions: 20 min/day, 1 daily session, 40 sessions/14 months - Montage: anode over left anterior temporal pole (FT7- FT9), cathode over right supraorbital location (FP2) - Electrodes size: anode 5 × 7 cm, cathode 5 × 7 cm - Intensity: 1.5 mA - tDCS device: NeuroConn	Semantic feature analysis therapy	No	2 and 4 weeks after every 10 sessions	- Oral naming of trained objects and actions - Oral naming of untrained objects and actions - RWT - BNT	None	↑ Oral naming of trained items ↑ Oral naming of untrained items ↑ BNT Gains maintained at **follow-ups**: ↑ Oral naming of untrained items	-
George et al., 2025 [126]	2 svPPA 2 lvPPA 6 mixed/unclassifiable PPA	70.0 (6.9); 6/4	Case series pre-post	- Sessions: 30 min/day, 1 daily at-home session, 20 sessions/4 weeks - Montage: anode over left IFG (F7), cathode over O1 - Electrodes size: anode 5 × 5 cm, cathode 5 × 5 cm - Intensity: 2 mA - At-home tDCS device: Soterix mini-CT	Individualised lexical retrieval treatment	No	None	- Oral naming of trained objects and actions - Oral naming of untrained objects and actions - QAB - BNT - Phonemic fluency - Semantic fluency - ACOM - Perception of change reported by the caregiver	- SAQOL-39 - PROMIS	↑ Oral naming of trained items ↑ BNT Perception of positive change reported by the caregiver	No significant improvements
Granadillo et al., 2025 [127]	4 lvPPA	69.0 (5.1); 2/2	Crossover RCT	- Sessions: 20 min/day, 1 daily session, 10 sessions/2 weeks - Montage: HD-tDCS, centre-surround 4 × 1 configuration, anode over left posterior SMG - Intensity: 2 mA - HD-tDCS device: MxN-9	Non-word repetition and word/non-word reading training	Yes	8 weeks post-treatment	- Repetition of trained non-words - Repetition of untrained non-words - Reading of trained words and non-words - Reading of untrained words and non-words - Rhyme matching of words and non-words - Oral picture naming from NAB - Phonemic fluency - Semantic fluency - Written words and sentence comprehension from NAB - Picture description from NAB	- Phonological short-term memory - Digit span - MoCA	↑ Repetition of trained non-words after placebo tDCS for a participant ↑ Reading of trained non-words after placebo tDCS for two participants Gains at **follow-up**: ↑ Repetition of trained non-words after placebo tDCS for a participant ↑ Repetition of trained non-words after anodal tDCS for a participant ↑ Reading of trained words after anodal tDCS for a participant ↑ Reading of trained non-words after placebo tDCS for a participant	No significant improvements
Nickels et al., 2025 [128]	3 nf/avPPA 9 lvPPA	70.7 (4.6); 3/9	Crossover RCT	- Sessions: 20 min/day, 1 daily session, 10 sessions/2 weeks - Montage: anode over left IFG or left SMG, cathode over right supraorbital location - Electrodes size: anode 5 × 7 cm, cathode 5 × 7 cm - Intensity: 1.5 mA - tDCS device: NeuroConn DC Stimulator Plus	Phonological treatment	Yes	8 weeks post-treatment	- Blending sounds for words and non-words -Reading non-words - Sound-letter transcoding from APB - Phonological manipulation from APB - Semantics Camel and Cactus test - Allographic conversion - Repetition of word and nonword - Reading words and non-words - Spelling words and non-words - Written narratives skills - BNT - Spontaneous speech, auditory-verbal comprehension, repetition, naming, reading, writing, apraxia and aphasia quotient from WAB - Perception of change reported by participants	- Digit span BW - Recognition of faces from Warrington memory test - MoCA - RCPM	↑ Blending sounds for words after Phase 1 of anodal tDCS-first group ↑ Blending sounds for non-words after both Phases of anodal tDCS-first and after Phase 1 of placebo tDCS- first group ↑ Reading non-words after Phase 1 of anodal tDCS-first group ↑ Sound-letter transcoding after Phase 1 of anodal tDCS-first and placebo tDCS-first groups ↑ Phonological manipulation after Phase 1 of anodal tDCS-first group ↑ Written narratives skills after Phase 1 of anodal tDCS-first group ↑ Perception of change reported by participants after Phase 1 and Phase 2 of anodal tDCS-first group and Phase 1 of placebo tDCS-first group Gains at **follow-up**: ↑ Blending sounds for words after anodal tDCS-first ↑ Blending sounds for non-words after placebo tDCS-first ↑ Sound-letter transcoding after anodal tDCS-first ↑ Reading non-words after anodal tDCS-first ↑ Spelling non-words after anodal tDCS-first ↑ Written narratives skills after anodal tDCS-first	Gains at follow-up: ↑ Recognition of faces after anodal tDCS-first group
Sheppard et al., 2025 [129]	3 nf/avPPA 2 lvPPA 3 svPPA	68.3 (5.7); 4/4	Crossover RCT	- Sessions: 20 min/day, 1 daily session, 15 sessions/3–5 weeks - Montage: anode over left IFG (F7), cathode over right shoulder - Electrodes size: anode 5 × 5 cm, cathode 5 × 5 cm - Intensity: 1–2 mA - tDCS device: Soterix 1 × 1 Clinical Trials	Verb Network-Strengthening Treatment, VNeST	Yes	8 weeks post-treatment	- Oral naming of trained verbs - Oral naming of untrained verbs - Oral naming of nouns - Sentence production from NAVS - Sentence comprehension from NAVS - Speech production (Cookie Theft picture from the BDAE)	None	↑ Oral naming of trained verbs in both conditions ↑ Speech production in both conditions Higher performance at oral naming of untrained verbs after anodal tDCS Higher performance at sentence production after anodal tDCS Gains maintained at **follow-up**: Higher performance at oral naming of untrained verbs after anodal tDCS Higher performance at sentence comprehension after anodal tDCS	-

↑: Statistically significant improvement; AAT: Aachener Aphasie Test; ACOM: Aphasia Communication Outcome Measure; ANT: Attention Network Test; AOS: Apraxia of speech; APB: Arizona Phonological Battery; ASRS: Aphasia Severity Rating Scale; BA: Broadmann Area; BADA: Battery for Analysis of Deficit Aphasic; BAT: Bilingual Aphasia Test; BDAE: Boston Diagnostic Aphasia Examination; BNT: Boston Naming Test; BW: Backward; cm: centimetres; DLPFC: Dorsolateral Prefrontal Cortex; HD-tDCS: High-Definition transcranial Direct-Current Stimulation; F: female; FW: Forward; IFG: Inferior Frontal Gyrus; inch: inches; IPL: Inferior Parietal Lobe; lvPPA: logopenic variant of Primary Progressive Aphasia; L1: Native language; L2: Second language; M: male; mA: milliAmpere; MMSE: Mini Mental State Examination; MoCA: Montreal Cognitive Assessment; n.a.: not available; NAB: Neuropsychological Assessment Battery; NAVS: Northwestern Assessment of Verbs and Sentences; nf/avPPA: non-fluent/agrammatic variant of Primary Progressive Aphasia; PHQ-9: Patient Health Questionnaire-9; PPA: Primary Progressive Aphasia; PROMIS: Patient-Reported Outcome Measurement Information System; QAB: Quick Aphasia Battery; RCPM: Raven’s Colored Progressive Matrices; RCT: Randomised Controlled Trial; ROCF: Rey–Osterrieth Complex Figure; RWT: Regensburg Word Fluency Test; SAQOL-39: Stroke and Aphasia Quality-of-Life Scale; SD: Standard Deviation; SMG: Supramarginal Gyrus; svPPA: semantic variant of Primary Progressive Aphasia; tDCS: transcranial Direct-Current Stimulation; TMT: Trail Making Test; VNeST: Verb Networking Strengthening Treatment; WAB: Western Aphasia Battery; WAB-R: Western Aphasia Battery-Revised.

**Table 4:** tDCS during verbal task without language treatment.

Study	Patients	Age: Mean (SD); Sex: M/F	Protocol Design	tDCS (Number of Sessions, Montage, and Parameters)	Language Treatment	Placebo	Follow-Up	Outcome Measures		Results	
								Language	Clinical and Cognitive	Language	Clinical and Cognitive
Gervits et al., 2016 [130]	2 nf/avPPA 4 lvPPA	66.2 (5.7); 1/5	Case series pre-post	- Sessions: 20 min/day, 1 daily session, 10 sessions/2 weeks - Montage: anode over left fronto-temporal region (F7), cathode over left occipito-parietal region (O1) - Electrodes size: anode 5 × 5 cm, cathode 5 × 5 cm - Intensity: 1.5 mA - tDCS device: Magstim Eldith	None (tDCS during narrative task)	No	6 and 12 weeks post-treatment	- Speech production - Sentence Repetition - Grammatical Comprehension - Semantic Processing (BNT; PPT; Semantic fluency) - Averaged language performance	None	↑ Speech production ↑ Grammatical Comprehension ↑ Averaged language performance Gains maintained at **follow-ups**: ↑ Grammatical Comprehension ↑ Averaged language performance	-
McConathey et al., 2017 [131]	6 nf/avPPA 1 lvPPA	68.7 (6.9); 2/5	Crossover RCT	- Sessions: 20 min/day, 1 daily session, 10 sessions/2 weeks - Montage: anode over left prefrontal region (F7), cathode over the left occipital region (O1) - Electrodes size: anode 5 × 5 cm, cathode 5 × 5 cm - Intensity: 1.5 mA - tDCS device: Magstim Eldith	None (tDCS during narrative task)	Yes	6 and 12 weeks post-treatment	- Repetition (sentence repetition from NACC-FTLD consortium) - Grammatical Comprehension (TROG) - Semantic Processing (BNT; PPT; Semantic fluency) - Averaged language performance	None	No significant improvements No significant improvements at **follow** **ups** Averaged language performance, Grammatical Comprehension, Semantic Processing: improvement after anodal tDCS in individuals who scored lower at baseline Speech repetition: improvement after anodal tDCS in individuals who scored higher at baseline	-
Hosseini et al., 2019 [132]	3 nf/avPPA 3 lvPPA	67.0 (10.6); 2/4	Crossover RCT	- Sessions: 20 min/day, 1 daily session, 10 sessions/2 weeks - Montage: anode over left prefrontal regions (F7), cathode over left occipital region (O1) - Electrodes size: anode 5 × 5 cm, cathode 5 × 5 cm - Intensity: 1.5 mA	None (tDCS during narrative task)	Yes	6 and 12 post-treatment	- Semantic fluency - Grammatical Comprehension (TROG) - PPT - BNT	None	↑ Semantic fluency after anodal tDCS Gains at **follow-ups**: ↑ Semantic fluency after anodal tDCS	-
Crowley et al., 2024 [133]	1 lvPPA	57.0; F	Single-case crossover	- Sessions: 20 min/day, 1 daily session, 30 sessions/8 weeks - Montage: HD-tDCS, anode over left TPJ, cathode over C1, CPz, Pz, Poz, O1, P9 or anode over left IFG, cathode over F9, FT7, FC1, F1, AFz, Fpz - Intensity: 4 mA - HD-tDCS device: MxN-9	None (tDCS during narrative task)	No	None	- Phonemic fluency - Semantic fluency - MINT - UDS 3.0 - Picture description	None	↑ MINT after anodal left TPJ tDCS ↑ Picture description after both conditions	-

↑: Statistically significant improvement; BNT: Boston Naming Test; cm: centimetres; HD-tDCS: High-Definition transcranial Direct-Current Stimulation; F: female; IFG: Inferior Frontal Gyrus; lvPPA: logopenic variant of Primary Progressive Aphasia; M: male; mA: milliAmpere; MINT: Multilingual Naming Test; NACC-FTLD: National Alzheimer’s Coordinating centre—FrontoTemporal Lobar Degeneration; nf/avPPA: non-fluent/agrammatic variant of Primary Progressive Aphasia; PPA: Primary Progressive Aphasia; PPT: Pyramids and Palm Trees test; RCT: Randomised Controlled Trial; SD: Standard Deviation; tDCS: transcranial Direct-Current Stimulation; TPJ: temporo-parietal junction; TROG: Test for the Reception of Grammar; UDS 3.0: Uniform Data Set 3.0.

## Abbreviations

The following abbreviations are used in this manuscript:

AAT	Aachener Aphasie Test
AD	Alzheimer’s Disease
BNT	Boston Naming Test
CAL	Communicative Activity Log
CBI	Cambridge Behaviour Inventory
CILT	Constraint-Induced Language Therapy
DLPFC	Dorsolateral Prefrontal Cortex
DMN	Default Mode Network
EEG	Electroencephalogram
fMRI	functional Magnetic Resonance Imaging
FTLD	Frontotemporal Lobar Degeneration
HD-tDCS	High-Definition transcranial Direct-Current Stimulation
Hz	Hertz
IFG	Inferior Frontal Gyrus
IPL	Inferior Parietal Lobe
lvPPA	logopenic variant of Primary Progressive Aphasia
L1	Native language
L2	Second language
LTP	Long-Term Potentiation
mA	Milliampere
MEG	Magnetoencephalography
nf/avPPA	non-fluent/agrammatic variant of Primary Progressive Aphasia
NPI	Neuropsychiatric Inventory
PET	Positron Emission Tomography
PFC	Prefrontal Cortex
PPA	Primary Progressive Aphasia
PPAOS	Primary Progressive Apraxia Of Speech
PPR	Posterior Perisylvian Region
RCT	Randomised Controlled Trial
rTMS	repetitive Transcranial Magnetic Stimulation
SAQOL-39	Stroke and Aphasia Quality-of-Life Scale-39
SLT	Speech and Language Therapy
SMG	Supramarginal Gyrus
svPPA	semantic variant of Primary Progressive Aphasia
tDCS	transcranial Direct-Current Stimulation
TPJ	Temporoparietal Junction
VNeST	Verb Network-Strengthening Treatment
